# Identification of Oxindoleacetic Acid Conjugates in Quinoa (*Chenopodium quinoa* Willd.) Seeds by High-Resolution UHPLC-MS/MS

**DOI:** 10.3390/molecules27175629

**Published:** 2022-08-31

**Authors:** Maarit Karonen, Juha-Matti Pihlava

**Affiliations:** 1 Natural Chemistry Research Group, Department of Chemistry, University of Turku, 20014 Turku, Finland; 2 Production Systems, Natural Resources Institute Finland (Luke), Myllytie 1, 31600 Jokioinen, Finland

**Keywords:** *Chenopodium quinoa*, indoleacetic acid, phenolic acids, phenolics, UHPLC-QOrbitrap, UHPLC-QTOF

## Abstract

Quinoa (*Chenopodium quinoa* Willd.) has a high nutritional value and it contains a high number and high amounts of specialized metabolites. These metabolites include, for example, phenolic acids, flavonoids, terpenoids and steroids. In addition, it is known to contain N-containing metabolites, such as betalains. Here, we report the presence and identification of 14 new oxindoleacetate conjugates in quinoa by high-resolution ultrahigh-performance liquid chromatography quadrupole-time-of-flight tandem mass spectrometry (UHPLC-QTOF-MS/MS) and ultrahigh-resolution UHPLC-QOrbitrap-MS/MS. The oxindoleacetate conjugates were extracted from dried and ground quinoa seeds using either methanol/water or acetone/water (4:1, *v*/*v*) and were further concentrated into aqueous phase and analyzed by UHPLC with reverse-phase chromatography using acetonitrile and 0.1% aqueous formic acid as eluents. High-resolution hybrid LC-MS/MS techniques, including full scan MS with in-source collision, induced dissociation, and data dependent-MS^2^(TopN) with stepped normalized collision energies using N_2_ as collision gas and data-independent acquisition (MS^E^) using ramped collision energies and argon as collision gas enabled their analysis directly from the crude quinoa seed extract. The oxindoleacetate conjugates were found to be present in both conventional and organic farmed seeds. According to our best knowledge, this is the first time hydroxy-oxindoles have been reported in quinoa.

## 1. Introduction

Quinoa (*Chenopodium quinoa* Willd.) is a pseudocereal originating from South America’s Andean regions. The main producers of quinoa are Bolivia and Peru, although quinoa is also cultivated in, e.g., North America, China and Europe [1], and even in Northern Europe [2,3,4]. Quinoa kernel is a one-seeded achene with the main structures being the seed coat (pericarp), curved embryo and perisperm. Perisperm, which makes up about 40% of the kernel volume, is mainly for starch storage, while the embryo and the small endosperm contains more proteins and lipids [5,6]. Kernels are relatively small, roughly 2 mm in diameter and with a thousand-seed weight between 1.90 and 3.68 g [2]. Seed shape can be lenticular, cylinder, ellipsoid or conical and the color variation can be from white to red to black [2,7]. The outer layer of the quinoa seed coat is also enriched with bitter saponins, and they must be removed by washing or abrasive milling prior to food use. Varieties with naturally low saponin content, so-called “sweet” varieties, have also been bred, especially in Denmark and the Netherlands [1,8].

**Figure 1:**
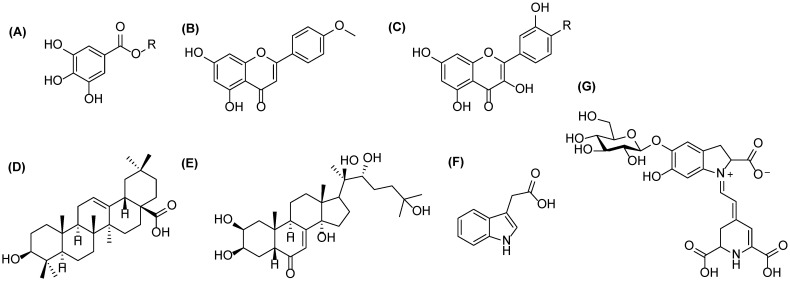
Examples of the metabolites presents in quinoa: (**A**) gallic acid (R = H) or galloylglucose (R = glc) for phenolic acids and their conjugates, (**B**) acacetin for flavones, (**C**) kaempferol (R = H) and quercetin (R = OH) for flavonols, (**D**) oleanolic acid for triterpenoids, (**E**) 20-hydroxyecdysone for phytoecdysteroids, (**F**) indole-3-acetic acid for plants hormones, and (**G**) betanin for betalains.

Quinoa has gained popularity around the world as a food because of its high nutritional value and because it is gluten-free [9]. Quinoa is also a good source of proteins: the bioactivities of quinoa protein hydrolysates and peptides have been of recent interest [10]. In addition, quinoa is known to contain a high amount of the specialized metabolites (previously defined as secondary metabolites), such as various phenolic acids, flavonols, flavanones, flavanols, betacyanins and especially phytoecdysteroids (Figure 1), which may have various beneficial health effects [8,11]. One of the most interesting primary metabolites in quinoa is indole acetic acid (IAA, Figure 1). It is an auxin class plant hormone, which has various regulatory functions in plant growth and development. In addition to plants, it is also produced by bacteria and fungi. IAA is catabolized to inactive forms, e.g., by oxidation, and it has been speculated that the oxidized IAA could have an important role in regulating bioactive auxin levels which in turn control the plant growth and morphogenesis [12]. Just recently, Hayashi et al. presented the major inactivation pathway for IAA which modulates auxin homeostasis in plant development in *Arabidopsis* [13]. Other oxidation products of IAA reported earlier are, for example, methyl-5-hydroxy-2-oxindole-3-acetic acid and its glycosides in rice (*Oryza sativa*) bran [14,15] and 7-hydroxy-2-oxindole-3-acetic acid 7′-O-*β*-D-glucoside in maize (*Zea mays*) seedlings [5].

Quinoa metabolites have been analyzed with many different analytical techniques. Traditional analytical HPLC-UV or HPLC-DAD have been applied for phenolic acids and flavonoids [16,17]. The detailed compositions of different forms of polyphenols and betacyanins have been obtained by HPLC or UHPLC combined with electrospray ionization mass spectrometry (ESI-MS) [18,19,20]. GC-MS profiling has been used for triterpenoid saponins [21] and headspace-GC -ion mobility MS for volatile compounds [22]. In addition, the purified polysaccharide fractions from quinoa has been analyzed by sugar composition, high-performance steric exclusion chromatographic, methylation and ^13^C NMR spectroscopic analyses [23]. We started our research by screening low-saponin quinoa samples by UHPLC-QTOF-MS/MS with the main focus being phenolic acids and their conjugates, flavonoids, phytoecdysteroids and saponins. While analyzing quinoa samples by UHPLC-QTOF, some new nitrogen containing compounds were observed for which no matching literature references were found. However, the resolution of TOF was not enough to obtain the unambiguous exact masses and corresponding molecular formulae. Therefore, more in-depth analysis and identification of these compounds in quinoa were conducted by UHPLC-QOrbitrap-MS/MS. These nitrogen-containing compounds were identified as methoxy- and hydroxyoxindole acetic acids and their various conjugates. 

## 2. Results and Discussion

MS and MS/MS data for quinoa metabolites were obtained by negative and positive electrospray ionization (ESI) and high-resolution mass analyzers and were reproduced by Waters MassLynx and Thermo Xcalibur software with the latter using the nitrogen-rule, mass tolerance of a maximum of 10 ppm, RDB equivalent between −1–100 and elements in use C 0–100, H 0–200, O 0–100 and N 0–10. The high-resolution data with exact masses and corresponding molecular formulae enabled the full characterization of both the precursor and product ions. The chemical composition of quinoa is well-known with at least 193 different specialized metabolites previously detected [11]. We also detected many of these known metabolites, such as phenolic acids, flavonoid aglycones and glucosides, for example, quercetin and rutin, and terpenoids, for example, hederagenin. Here, we focus on the new metabolites having a surprisingly interesting composition.

**Figure 2:**
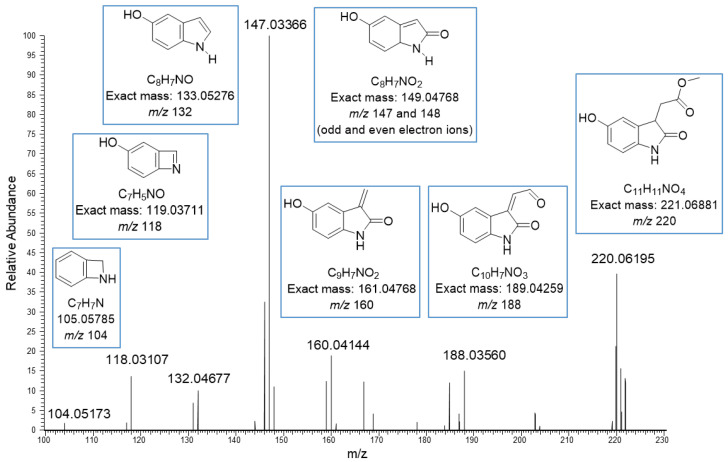
Proposed MS/MS fragmentation for methyl-5-hydroxyoxindole-3-acetate obtained by negative ion mode using electrospray ionization high-resolution tandem mass spectrometry. The ions are deprotonated.

The basic core in the new structures seemed to be methyl-5-hydroxyoxindole-3-acetate (MeO-oxIAA) having the [M–H]^−^ ion at *m*/*z* 220.06195 corresponding to the exact mass 221.06881 Da ja the molecular formula C_11_H_11_O_4_N. This kind of structure has been previously detected in rice bran by Kinashi et al. [14]. The structure of MeO-oxIAA was confirmed by its MS/MS fragmentation as shown in Figure 2. The data show nicely the fragmentation of the acetate side chain: *m*/*z* 188 corresponds to the cleavage of methoxy group, *m*/*z* 160 to the cleavage of the carbonyl and methoxy groups and *m*/*z* 148 and 147 to the loss of the whole side chain. The ions at both *m*/*z* 147 and *m*/*z* 148 indicate the presence of both odd and even electron fragment ions, which is fully possible as stepped normalized collision energies at 20, 50 and 80 eV were used. The rest of the product ions at *m*/*z* 132, 118 and 104 correspond to the fragmentation of the heterocyclic dihydropyrrolone ring. A similar fragmentation pattern was obtained by positive ionization. Nonhebel and Bandurski have reported a similar kind of IAA derivative from *Zea mays* seedlings [24]. In these IAA derivatives, the hydroxyl substituent was in another position, i.e., the oxIAA was hydroxylated in 7-position (7-OH-oxIAA). Based on the mass spectral data, it is impossible to determine the exact position of the hydroxyl group. In general, MeO-oxIAA is a methyl ester of 5-hydroxyoxindole-3-acetate (OH-OxIAA), which is the derivative of IAA and is considered to be the key intermediate in the conversion of indoles to quinolone structures [14].

**Figure 3:**
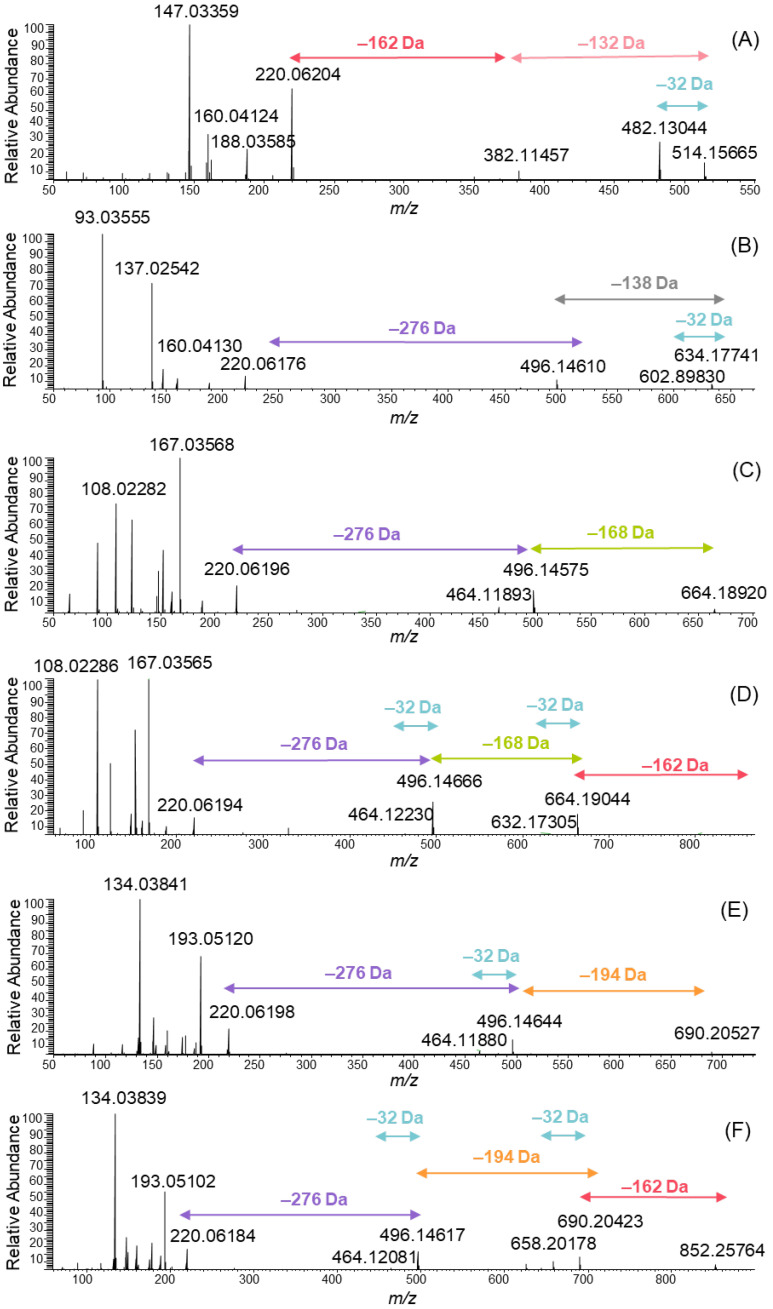
MS/MS spectra of the ions at *m*/*z* (**A**) 514, (**B**) 634, (**C**) 664, (**D**) 826, (**E**) 690 and (**F**) 852 obtained by negative electrospray ionization high-resolution tandem mass spectrometry.

A series of MeO-oxIAA derivatives were detected as shown in Figure 3. The MS/MS spectra of these derivatives were obtained from data-dependent MS/MS total ion chromatograms using large retention time windows in order to obtain a higher average number of data points: *m*/*z* 514 was obtained from the retention time of 2.51–2.58 min, 634 from 3.06–3.62 min, 664 from 3.14–3.76 min, 826 from 3.15–3.19 min, 690 from 3.45–4.41 and 852 from 3.48–4.02 min. All MS/MS spectra clearly showed the presence of MeO-oxIAA by the fragment ion at *m*/*z* 220. In addition, the mass difference of 32 Da corresponding to the cleavage of the methoxy group from the acetate side chain was evidenced for each derivative. The derivatives having molar masses of 383 and 515 Da eluted first and were the most polar ones exhibiting two sugar moieties attached to MeO-oxIAA. The polarity of the compounds was estimated based on their retention order in the reversed-phase chromatography used. The sugar units attached to the derivative with a molar mass of 515 Da were hexose (C_6_H_12_O_6_) and pentose (C_5_H_10_O_5_) exhibited the characteristic fragments of 162 Da and 132 Da, respectively (Figure 3A). 

Similarly, one hexose was found to be attached to the derivative that had a molar mass of 383 Da. The oxIAA derivatives have been previously found to be glycosylated; for example, in *Zea mays* seedlings, IAA is known to transform to oxIAA and then to OH-oxIAA and finally to OH-oxIAA-glucoside [24], also detected in rice bran [15]. The hexose moiety was detected by the neutral cleavage of 162 Da in many MeO-oxIAA derivatives (Figure 3A,D,F). In addition to separate hexose and pentose, a loss of the sugar unit producing a mass difference of 276 Da was detected (Figure 3B–F). This mass difference corresponds to the elemental composition of C_11_H_16_O_8_. Typically, the glycosylation is *O*-glycosylation, i.e., the sugar is attached to the oxygen atom of the phenolic hydroxyl group. The loss of C_11_H_18_O_9_ indicates the presence of hexose-pentose disaccharide [25,26]. The difference between the C_11_H_16_O_8_ observed and the C_11_H_18_O_9_ found in the literature is H_2_O, which indicates that the disaccharide is attached to another monosaccharide with an additional ester bond. This is in agreement with the previous data, as the non-starch polysaccharides in quinoa are known to contain branched structures and the main neutral sugars being arabinose, rhamnose and galactose [23]. The other option for the sugar unit with the elemental composition of C_11_H_18_O_9_ could be acylated hexose recently reported by Baky et al. from African baobab fruits [27]. However, in this case the MS/MS data better support the presence of branched trisaccharide.

In addition to sugar units, the MeO-oxIAA derivatives contained phenolic acid moieties. Hydroxybenzoic acid is present in the spectrum in Figure 3B, vanillic acid in Figure 3C and the corresponding glycoside in Figure 3D, and ferulic acid in Figure 3E and the corresponding glycoside in Figure 3F. Hydroxybenzoic acid exhibited an [M–H]^−^ ion at *m*/*z* 137.02542 corresponding to the elemental composition of C_7_H_6_O_3_ of the initial molecule and a fragment ion at *m*/*z* 93.0355 corresponding to the cleavage of the carboxyl group (Figure 3B). In addition, a neutral cleavage of 138 Da from the MeO-oxIAA derivative was detected. Similar observations were made for vanillic acid (an ion at *m*/*z* 167 and the neutral cleavage of 168 Da; Figure 3C,D) and for ferulic acid (an ion at *m*/*z* 193 and the neutral cleavage of 194 Da; Figure 3E,F).

**Figure 4:**
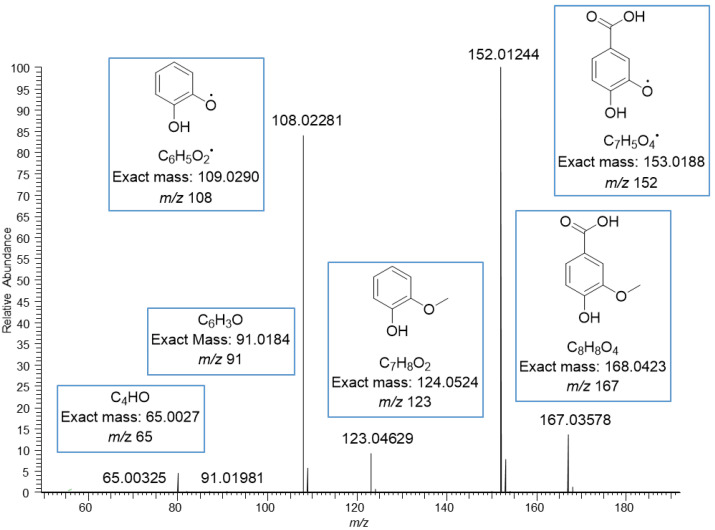
MS/MS fragmentation of vanillic acid moiety obtained by negative ionization using electrospray high-resolution tandem mass spectrometry. The ions at *m*/*z* 108, 123, 152 and 167 are deprotonated.

**Figure 5:**
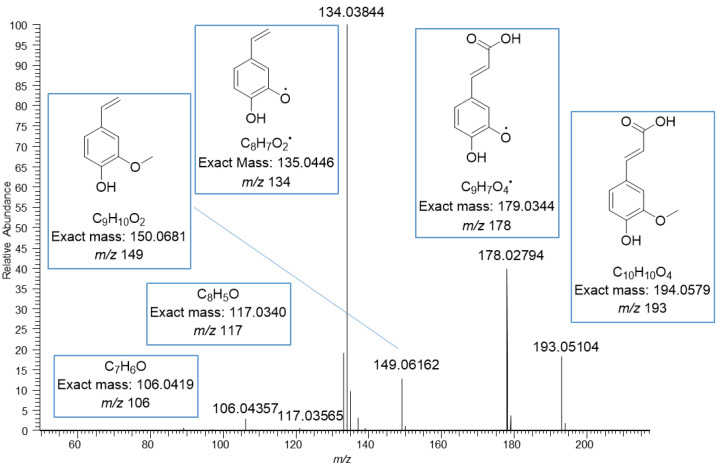
MS/MS fragmentation of ferulic acid moiety obtained by negative ionization using electrospray high-resolution tandem mass spectrometry. The ions at *m*/*z* 134, 149, 178 and 193 are deprotonated.

The MS/MS spectrum of vanillic acid exhibited the fragment ions at *m*/*z* 152 corresponding to the loss of methyl group, at *m*/*z* 123 corresponding to the loss of carboxyl group, at *m*/*z* 108 corresponding to the losses of both methyl and carboxyl groups, and at *m*/*z* 91 and 65 resulting from the fragmentation of the phenolic ring (Figure 4). The ions in the mass spectrum were exactly the same as the ions in the ESI-MS/MS product scan of vanillic acid obtained using 30 eV collision energy by Grieman et al. [28]. The MS/MS spectrum of ferulic acid exhibited a very similar fragmentation pattern to the vanillic acid with the mass difference of 26 Da due to the different side chain, i.e., carboxyl group in vanillic acid and phenyl propenoic in ferulic acid. The spectrum of ferulic acid exhibited the fragment ions at *m*/*z* 178, corresponding to the loss of the methyl group, at *m*/*z* 149 corresponding to the loss of the carboxyl group, at *m*/*z* 134 corresponding to the losses of both methyl and carboxyl groups, and at *m*/*z* 117 and 106 for the phenolic ring with the side chain (Figure 5). Similar fragments have been observed by He et al. and Balasoiu et al. [29,30]. All these detected phenolic acids are well-known constituents in quinoa; for example, Gawlik-Dziki et al. have reported the contents of *p*-hydroxybenzoic acid, vanillic acid and ferulic acid to be in quinoa leaf extracts 10, 23, and 762 µg/g DW, respectively [31]. Repo-Carrasco-Valencia et al. [16] reported the contents of phenolic acids as a sum of free, soluble conjugated and insoluble bound forms in ten quinoa seed samples. The amount of total ferulic acid varied between 120.0–200.0 µg/g, vanillic acid 89.7–146.0 µg/g, *p*-coumaric acid 22.6–275.0 µg/g, *p*-hydroxybenzoic acid 19.2–38.8 µg/g and caffeic acid 2.5–14.7 µg/g. The part of free and soluble conjugated forms compared to the total amount varied as follows: ferulic acid 3–52%, vanillic acid 10–60%, *p*-coumaric acid 0–72%, *p*-hydroxybenzoic acid 55–98% and caffeic acid 0–98% [16]. 

Tang et al. found the contents of *p*-hydroxybenzoic acid to vary between 15.8 and 17.2 µg/g in the seeds of white, red and black quinoa species [18]. Similarly, the contents of vanillic acid and its 4-glucoside varied between 39.0 and 70.0 and between 23.1 and 27.4 µg/g and of ferulic acid and its 4-glucoside between 37.5 and 58.4 and 132.0 and 161.4 µg/g, respectively [18]. In addition to above mentioned phenolic acids, *p*-coumaric acid derivatives, such as *p*-coumaric acid glycoside, have been found in red and black quinoa seeds, but not in white [18]. We did not detect any *p*-coumaric acid conjugates within this study, but it could be possible that the pigmented quinoas would also contain them as oxIAA conjugates. 

**Figure 6:**
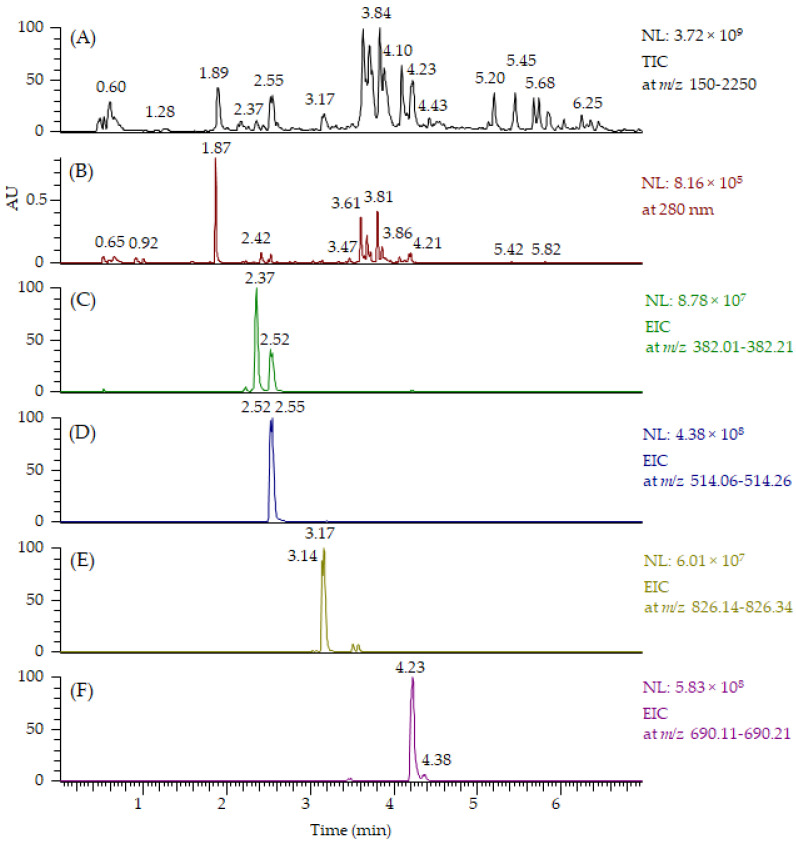
(**A**) Total ion chromatogram (TIC) at *m*/*z* 150–2250 (TIC) and (**B**) UV chromatogram at 280 nm obtained by UHPLC-QOrbitrap-MS/MS showing all compounds present in the quinoa seed extract. Extracted ion chromatograms (EICs) of the selected oxindoleacetic acid conjugates present: (**C**) **3** at *m*/*z* 382, (**D**) **4** at *m*/*z* 514, (**E**) **7** at *m*/*z* 826 and (**F**) **14** at *m*/*z* 690. Bold numbers refer to Table 1. AU = absorbance unit, NL = normalized intensity.

All MeO-oxIAA derivatives detected by UHPLC-QOrbitrap-MS/MS are presented in Table 1. For some of the derivatives, two isomers at different retention times were detected. In addition to MeO-oxIAA conjugates, we found similar derivatives based on the OH-oxIAA core. These OH-oxIAA conjugates were similarly glycosylated and also contained phenolic acid moieties in their structures (Table 1). The corresponding data obtained by UHPLC-QTOF-MS/MS are presented in Supplementary material in Tables S1 and S2. The fragmentation patterns of OH-oxIAA derivatives were also similar, showing the losses of sugar units and phenolic acid moieties. This time, two kinds of fragments were noticed for the cleavage of phenolic acids: these corresponded to the cleavage of the free vanillic acid group (−168 Da), but also for the loss of vanilloyl group (−150 Da) and, likewise, for free ferulic acid (−194 Da) and for the feruloyl group (−176 Da). The composition of oxIAAs and their conjugates were found to be similar in both the conventional and organic farmed low-saponin quinoa samples. UHPLC-MS/MS proved to be an efficient technique providing chromatograms with distinct peaks, and oxIAA derivatives could be nicely separated from the other compounds presented by MS (Figure 6). Figure 6A,B exhibit the total ion chromatogram (TIC) and UV chromatogram obtained by UHPLC-QOrbitrap-MS/MS for all compounds present, respectively. Extracted ion chromatograms (EICs) at *m*/*z* 382 for MeO-oxIAA hexose (Figure 6C), at *m*/*z* 514 for MeO-oxIAA hexose-pentose (Figure 6D), at *m*/*z* 826 for MeO-oxIAA hexose-hexose-pentose conjugated with vanillic acid (Figure 6E) and at *m*/*z* 690 for MeO-oxIAA hexose-pentose conjugated with ferulic acid (Figure 6F) shows the peak performance and the presence of isomers. However, it must be noted that EICs also show the presence of fragment ions, i.e., the peak with the retention time of 2.52 min in EIC at *m*/*z* 382 in Figure 6C corresponds to the fragment ion of *m*/*z* 514. In addition, it can be seen that the oxIAA derivatives with phenolic moieties (Figure 6E,F) eluted later than ones without (Figure 6C,D) and that the oxIAA derivatives with vanillic acid (Figure 6E) eluted earlier than the ones with ferulic acid (Figure 6F), which is in accordance with the literature regarding the elution order of vanillic and ferulic acids [18]. In general, OH-oxIAA derivatives eluted earlier than the corresponding MeO-oxIAA derivatives (see the retention times in Table 1, Tables S1 and Table S2) as the methylation decreased the polarity of the compounds. In this qualitative study, we did not evaluate the matrix effects of other co-eluting specialized metabolites present as the focus was on the characterization but, in general, the oxIAA derivatives ionized nicely with relatively high intensities (Figure 6C,D). However, it is fully possible that the other compounds can decrease the response of oxIAA conjugates (ion suppression) or increase their response (ion enhancement) and, therefore, the matrix effects should be later evaluated if a quantitative LC-MS method is developed for oxIAA conjugates.

OxIAAs are considered to be catabolic derivates of IAA [12,13,14,15,24]. Based on our results, the catabolic products of IAA, at least in *Chenopodium quinoa* seeds, form a much more complex group of oxIAAs and their derivatives than, for instance, in *Arabidopsis* [13], *Oryza sativa* [14,15] and *Zea mays* [24]. The actual amounts of oxIAAs in quinoa seeds are so far unclear and, also whether they are affected by, e.g., cooking or not and, finally, whether these compounds possess any biological activity when ingested. To ensure that the found oxIAA compounds were not limited to this particular low-saponin quinoa cultivar, our preliminary UHPLC-QTOF data were re-evaluated. As a result, these compounds were found in all quinoa seed samples analyzed over the years in our laboratory, e.g., in two black seeded and four white seeded Peruvian quinoa samples in the study by Repo-Carrasco-Valencia et al. [32] and in the light- to dark-brownish colored Finnish quinoa by Mattila et al. [3]. OxIAA compounds were also found in the abrasive milled seed sample [3], although their intensity was about 25% lower than in the corresponding non-milled sample (data not shown). However, more careful fractionation of the quinoa seed would be needed to conclude whether the oxIAAs are located in some specific part of the seed or not. 

## 3. Materials and Methods

### 3.1. Pre-Treatment and Extraction of the Plant Material

Conventional and organic farmed seed samples of low-saponin cultivar of quinoa were provided by GreenFood50 (Wageningen, The Netherlands). Both quinoas had been cultivated in the Netherlands. For preliminary screening test, the seeds were milled using a KT-120 hammer mill with an ø1 mm sieve (Koneteollisuus Oy, Klaukkala, Finland). For the preliminary screening test, 5.0 g of dry flour was extracted twice with methanol/water (4:1, *v*/*v*; 50 mL) for 30 min at RT using a magnetic stirrer. Then, samples were centrifuged (10 min; 600 g), and the combined supernatants were evaporated in a rotary evaporator to dryness under reduced pressure. Samples were dissolved in methanol/water (4:1, *v*/*v*; 2.0 mL), filtered through PTFE membrane filters (0.2 µm, Pall Corporation, Port Washington, NY, USA), and analyzed by UHPLC-QTOF.

For the final identification work, the seed material was ground into fine powder and 10 mg was extracted with mixed with 1.4 mL of acetone/water (4:1, *v*/*v*) and macerated overnight. Then, the samples were extracted twice with a similar volume of acetone/water (4:1, *v*/*v*), the extracts were combined and concentrated into aqueous phase and freeze-dried. Prior to the UHPLC-QOrbitrap-MS/MS analyses, the extracts were dissolved in water and were filtered with PTFE filters (0.2 µm, VWR International, Radnor, PA, USA).

### 3.2. UHPLC-MS/MS Analyses

UHPLC-MS/MS analyses were performed in two different laboratories using two different extraction methods and two different high-resolution MS instruments in order to ensure that the data and results were consistent, accurate and precise. Preliminary screening was performed by an Acquity UPLC-Xevo G2 QTOF high-resolution mass spectrometer (Waters, Milford, MA, USA) operated by Waters MassLynx 4.1 software was used for the screening tests. Analytical conditions were as previously described [33] except that the analytical column used was Waters Acquity HSS T3 (1.8 µm, 2.1 mm x 100 mm). Briefly, the column over temperature was 45 °C, acetonitrile and 0.1% HCOOH were used as eluents with a flow rate of 0.55 mL/min and an injection volume of 2 µL. The samples were analyzed on both positive and negative mode by data independent acquisition (MS^E^) centroid data mode in a full scan *m*/*z* 50–1500 with 0.2 sec scan time with argon as a collision gas. In the MS^E^ function, the precursor ions from the low-collision energy MS-mode were fragmented using high collision energy ramped up from 25 to 45 V [33]. 

The final identification was obtained by UPLC-QOrbitrap-MS/MS, performed as previously reported [34]. In short, the methodology utilized a reversed phase chromatography combined with ESI. Chromatographic conditions included a Waters Acquity BEH phenyl column (2.1 × 100 mm, 1.7 µm), acetonitrile and 0.1% HCOOH as eluents, flow rate of 0.5 mL/min, injection volume of 5 µl and diode array detection at 190–500 nm. The MS data was obtained by both negative and positive ionization using in-source collision-induced dissociation of 30 eV and stepped normalized collision energies of 20, 50 and 80 eV in the collision cell. The methods included full-scan MS with the mass range of *m*/*z* 150–2250 and MS/MS analyses with the dd-MS^2^(TopN) technique (N is the maximum number of ions to trigger after one survey scan and it was set at 3). The resolution of full-scan MS analysis was set at 35,000 and the resolution of dd-MS^2^(Top3) analyses at 17,500 corresponding to the transient length of 64 and 128 ms, respectively, allowing the collection of a sufficient number of MS and MS/MS data points in combination with fast chromatography used. The data were processed with Thermo Xcalibur software, version 4.1.31.9 (Thermo Fisher Scientific Inc., Waltham, MA, USA). The calculated exact masses (M_calculated_) were obtained using the following monoisotopic masses: 12.000000 (C), 1.007825 (H), 15.994915 (O) and 14.003074 (N) and the mass errors by the following equation:

(1) $$\mathrm{error}\;\left(\mathrm{ppm}\right)=\frac{M_{\mathrm{Measured}}-M_{\mathrm{Calculated}}}{M_{\mathrm{Calculated}}}\times 100,0000.$$

## 4. Conclusions

In this study, we detected new oxIAA conjugates in quinoa seeds using high-resolution UHPLC-MS/MS. Ultrahigh-resolution MS and MS/MS were used to measure the exact masses and the corresponding molecular formulae of these oxIAA conjugates but also to reveal their characteristic fragmentation patterns. Previous studies have shown that quinoa contains a high number of different specialized metabolites including, for example, phenolic compounds, terpenoids and steroids. The new compounds contained MeO-oxIAAs or OH-oxIAAs conjugated with sugars as evidenced by the characteristic neutral losses of 132, 162 and 276 Da and with hydroxybenzoic acid, vanillic acid or ferulic acid moieties supported by the characteristic product ions at *m*/*z* 137, 167 and 193 and the neutral cleavages of 138, 168 and 194 Da, respectively. The MS/MS techniques used, utilizing data dependent-MS^2^(TopN) with stepped normalized collision energies and data independent acquisition (MS^E^) using ramped collision energies and combined with UHPLC and ESI, allowed the fast analysis of the whole series of 14 different MeO-oxIAA and OH-oxIAA conjugates directly from the crude quinoa seed extract. 

## Figures and Tables

**Table 1:** Putative structure for the novel methyl-5-hydroxyoxindole-3-acetate (MeO-oxIAA, R_1_ = CH_3_) and 5-hydroxyoxindole-3-acetate (OH-oxIAA, R_1_ = H) glycosides having phenoyl substituents with their UPLC retention times (min), molecular formula, measured exact masses (based on the [M–H]^−^ ions), calculated exact masses, mass errors (ppm), ring and double bond equivalents (RDB), exact masses of the [M–H]^−^ ions and MS/MS fragment ions. The MS/MS fragments were not obtained for the minor derivatives (n/a, not available). R_2_ consist of the following structural units attached together by several ester bonds: FA (ferulic acid), hex (hexose), HBA (hydroxybenzoic acid), pent (pentose), and VA (vanillic acid).

									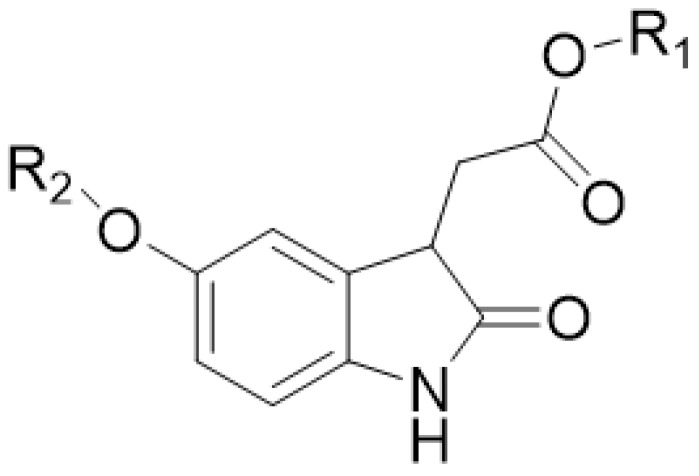
#	RT (min)	Name	Molecular Formula	M_measured_	M_calculated_	Error (ppm)	RDB	[M–H]^−^	Characteristic MS/MS Fragments in Negative Ionization
1	1.92	OH-oxIAA hex	C_16_H_19_O_9_N	369.10638	369.10598	1.1	8	368.09910	n/a
2	2.14; 2.18	OH-oxIAA hex-pent	C_21_H_27_O_13_N	501.14892	501.14824	1.3	9	500.14164	368, 206, 188, 162, 160, 147
3	2.37	MeO-oxIAA hex	C_17_H_21_O_9_N	383.12062	383.12163	−2.7	8	382.11334	350, 220, 188, 160, 146
4	2.52; 2.55	MeO-oxIAA hex-pent	C_22_H_29_O_13_N	515.16408	515.16389	0.4	9	514.15680	382, 220, 188, 160, 147
5	2.80	OH-oxIAAe VA hex-hex-pent	C_35_H_43_O_21_N	813.23403	813.23276	1.6	15	812.22675	650, 500, 482, 350, 206, 188, 162, 160, 147
6	3.06; 3.10	MeO-oxIAA HBA hex-hex-pent	C_35_H_43_O_20_N	797.24008	797.23785	2.8	15	796.23280	n/a
7	3.14; 3.17	MeO-oxIAA VA hex-hex-pent	C_36_H_45_O_21_N	827.24855	827.24841	0.2	15	826.24127	664, 496, 464, 329, 220, 188, 167, 160,
									152, 147, 123, 108, 91, 65
8	3.17; 3.22	OH-oxIAA HBA hex-pent	C_28_H_31_O_15_N	621.17018	621.16937	1.3	14	620.16290	482, 206, 188, 162, 160, 147
9	3.27; 3.31	OH-oxIAA VA hex-pent	C_29_H_33_O_16_N	651.18127	651.17994	2.0	14	650.17399	500, 482, 350, 206, 188, 167, 162, 160, 147
10	3.36; 3.48	MeO-oxIAA FA hex-hex-pent	C_38_H_47_O_21_N	853.26530	853.26406	1.4	16	852.25802	690, 496, 464, 276, 220, 193, 188, 178, 160,
									149, 147, 134, 117, 106
11	3.60; 3.66	MeO-oxIAA HBA hex-pent	C_29_H_33_O_15_N	635.18544	635.18500	0.7	14	634.17816	496, 220, 188, 160, 147, 137, 93
12	3.68; 3.73	MeO-oxIAA VA hex-pent	C_30_H_35_O_16_N	665.19628	665.19559	1.0	14	664.18900	496, 464, 276, 220, 188, 167, 160, 152, 147,
									123, 108, 91, 65
13	3.81	OH-oxIAA FA hex-pent	C_31_H_35_O_16_N	677.19599	677.19559	0.6	15	676.18871	500, 482, 350, 206, 193, 188, 162, 160, 147
14	4.23; 4.38	MeO-oxIAA FA hex-pent	C_32_H_37_O_16_N	691.21257	691.21124	1.9	15	690.20529	496, 464, 276, 220, 193, 188, 178, 160, 149,
									147, 134, 117, 106

## Data Availability

The data presented in this study are available on request from the authors.

## Supplementary Materials

The following supporting information can be downloaded at: https://www.mdpi.com/article/10.3390/molecules27175629/s1, Table S1. OTOF-data for the methyl-5-hydroxyoxindole-3-acetate (MeO-oxIAA) and 5-hydroxyoxindole-3-acetate (OH-oxIAA) glycosides having phenoyl substituents with their retention times (min), elemental composition, measured [M–H]^−^ ions, calculated [M–H]^−^ ions, error (ppm), characteristic MS/MS fragment ions obtained by negative ionization. FA (ferulic acid), hex (hexose), HBA (hydroxybenzoic acid), mal (malonate), *n*/a, not available, pent (pentose), tr (trace peak), and VA (vanillic acid). Table S2. OTOF-data for the methyl-5-hydroxyoxindole-3-acetate (MeO-oxIAA) and 5-hydroxyoxindole-3-acetate (OH-oxIAA) glycosides having phenoyl substituents with their retention times (min), elemental composition, measured [M–H]^−^ ions, calculated [M–H]^−^ ions, error (ppm), characteristic MS/MS fragment ions obtained by positive ionization. FA (ferulic acid), hex (hexose), HBA (hydroxybenzoic acid), mal (malonate), *n*/a, not available, pent (pentose), tr (trace peak), and VA (vanillic acid).

## Sample Availability

Samples of the compounds of quinoa material are not available from the authors.
