# Supplementary material for: Identification of Oxindoleacetic Acid Conjugates in Quinoa (Chenopodium quinoa Willd.) Seeds by High-Resolution UHPLC-MS/MS
Source: Molecules. 2022 Aug 31;27(17):5629. doi: 10.3390/molecules27175629 (PMC9458244; doi:10.3390/molecules27175629)
Supplement: Supplementary file 1 [file molecules-27-05629-s001.zip › molecules-1819873-supplementary.pdf]

Supplementary Material

Identification of oxindoleacetic acid conjugates in quinoa (*Chenopodium quinoa* Willd.) seeds by high-resolution UHPLC-MS/MS

Maarit Karonen[1,*] and Juha-Matti Pihlava [2,*]

[1] Natural Chemistry Research Group, Department of Chemistry, University of Turku, 20014 Turku, Finland
[2] Production Systems, Natural Resources Institute Finland (Luke), Myllytie 1, 31600 Jokioinen, Finland
* Correspondence: maarit.karonen@utu.fi (M.K.), juha-matti.pihlava@luke.fi (J-M.P.); Tel.: +358-29-4503179 (M.K.)

Table of Contents

Table S1. OTOF-data for the methyl-5-hydroxyoxindole-3-acetate (MeO-oxIAA) and 5-hydroxyoxindole-3-acetate (OH-oxIAA) glycosides having phenoyl substituents with their retention times (min), elemental composition, measured [M–H]⁻ ions, calculated [M–H]⁻ ions, error (ppm), characteristic MS/MS fragment ions obtained by negative ionization. FA (ferulic acid), hex (hexose), HBA (hydroxybenzoic acid), mal (malonate), n/a, not available, pent (pentose), tr (trace peak), and VA (vanillic acid). **2**

Table S2. OTOF-data for the methyl-5-hydroxyoxindole-3-acetate (MeO-oxIAA) and 5-hydroxyoxindole-3-acetate (OH-oxIAA) glycosides having phenoyl substituents with their retention times (min), elemental composition, measured [M–H]⁻ ions, calculated [M–H]⁻ ions, error (ppm), characteristic MS/MS fragment ions obtained by positive ionization. FA (ferulic acid), hex (hexose), HBA (hydroxybenzoic acid), mal (malonate), n/a, not available, pent (pentose), tr (trace peak), and VA (vanillic acid). **4**

Table S1. OTOF-data for the methyl-5-hydroxyoxindole-3-acetate (MeO-oxIAA) and 5-hydroxyoxindole-3-acetate (OH-oxIAA) glycosides having phenoyl substituents with their retention times (min), elemental composition, measured [M–H]⁻ ions, calculated [M–H]⁻ ions, error (ppm), characteristic MS/MS fragment ions obtained by negative ionization. FA (ferulic acid), hex (hexose), HBA (hydroxybenzoic acid), mal (malonate), n/a, not available, pent (pentose), tr (trace peak), and VA (vanillic acid).

RT (min)	Name	Elemental composition [M-H]⁻	Measured [M-H]⁻	Calculated [M-H]⁻	Error (ppm)	Characteristic MS/MS fragments in negative ionization
2.87	OH-oxIAA hex	C16H20NO9	368.0972	368.0982	-2.7	206, 162, 160
3.97	OH-oxIAA hex-pent	C21H26NO13	500.1406	500.1404	0.4	368, 206, 162, 160
4.09	OH-oxIAA hex-pent	C21H26NO13	500.1389	500.1404	-3.0	368, 206, 162, 160
4.32	MeO-oxIAA hex	C17H20NO9	382.1128	382.1138	-2.6	220, 206, 188, 160, 147, 132
4.81	MeO-oxIAA hex-pent	C22H28NO13	514.1558	514.1561	-0.6	482, 382, 350, 323, 282, 220, 206, 188, 162
4.89	MeO-oxIAA hex-pent	C22H28NO13	514.1562	514.1561	0.2	482, 382, 350, 323, 282, 220, 206, 188, 162
6.24	MeO-oxIAAe VA hex-hex-pent	C36H44NO21	826.2406	826.2406	0.0	664, 556, 524, 496, 167
6.32	MeO-oxIAAe VA hex-hex-pent	C36H44NO21	826.2400	826.2406	-0.7	664, 556, 524, 496, 167
6.41	OH-oxIAA HBA hex-pent	C28H30NO15	620.1614	620.1615	-0.2	500, 206, 162
6.50	OH-oxIAA HBA hex-pent	C28H30NO15	620.1635	620.1615	3.2	500, 206, 162
6.59	OH-oxIAA VA hex-pent	C29H32NO16	650.1724	650.1721	0.5	500, 482, 350, 206, 162, 160
6.70	OH-oxIAA VA hex-pent	C29H32NO16	650.1716	650.1721	-0.8	500, 482, 350, 206, 162, 160
6.96	MeO-oxIAAe FA hex-hex-pent	C38H46NO21	852.2595	852.2562	3.9	826, 788, 739, 690, 220, 193
7.06	MeO-oxIAAe FA hex-hex-pent	C38H46NO21	852.2568	852.2562	0.7	826, 788, 739, 690, 220, 193
7.21	MeO-oxIAA HBA hex-pent	C29H32NO15	634.1780	634.1772	1.3	496, 220, 167, 137
7.34	MeO-oxIAA HBA hex-pent	C29H32NO15	634.1782	634.1772	1.6	496, 220, 167, 137
7.36	MeO-oxIAA VA hex-pent	C30H34NO16	664.1895	664.1878	2.6	496, 220, 167
7.48	MeO-oxIAA VA hex-pent	C30H34NO16	664.1895	664.1878	2.6	496, 220, 167
7.65 (tr)	MeO-oxIAA VA hex-pent	C30H34NO16	664.1884	664.1878	0.9	n/a

7.71 (tr)	MeO-oxIAA VA hex-pent	C30H34NO16	664.1881	664.1878	0.5	n/a
7.83	OH-oxIAA FA hex-pent	C31H34NO16	676.1885	676.1878	1.0	206, 193, 162
8.56	MeO-oxIAA FA hex-pent	C32H36NO16	690.2048	690.2034	2.0	496, 220, 193, 134
8.63	MeO-oxIAA FA hex-pent	C32H36NO16	690.2043	690.2034	1.3	496, 220, 193, 134
8.80 (tr)	MeO-oxIAA FA hex-pent	C32H36NO16	690.2036	690.2034	0.3	496, 220, 193, 134
8.87 (tr)	MeO-oxIAA FA hex-pent	C32H36NO16	690.2037	690.2034	0.4	496, 220, 193, 134
9.31	MeO-oxIAA FA hex-pent-mal	C35H38NO19	776.2050	776.2038	1.5	732, 220, 193, 134
9.36	MeO-oxIAA FA hex-pent-mal	C35H38NO19	776.2036	776.2038	-0.3	732, 220, 193, 134
9.74	unknown MeO-oxIAA conjugate	C32H40NO16	694.2346	694.2347	-0.1	496, 220, 153
9.82	unknown MeO-oxIAA conjugate	C32H40NO16	694.2336	694.2347	-1.6	496, 220, 153
10.32	unknown MeO-oxIAA conjugate	C37H46NO16	760.2811	760.2817	-0.8	482, 263, 220, 153
10.39	unknown MeO-oxIAA conjugate	C37H46NO16	760.2811	760.2817	-0.8	482, 263, 220, 153

Table S2. OTOF-data for the methyl-5-hydroxyoxindole-3-acetate (MeO-oxIAA) and 5-hydroxyoxindole-3-acetate (OH-oxIAA) glycosides having phenoyl substituents with their retention times (min), elemental composition, measured [M–H]⁻ ions, calculated [M–H]⁻ ions, error (ppm), characteristic MS/MS fragment ions obtained by positive ionization. FA (ferulic acid), hex (hexose), HBA (hydroxybenzoic acid), mal (malonate), n/a, not available, pent (pentose), tr (trace peak), and VA (vanillic acid).

RT (min)	Name	Elemental composition $[M+H]^+$	Measured [M+H]+	Calculated $[M+H]^+$	Error (ppm)	Characteristic MS/MS fragments in positive ionization
2.87	OH-oxIAA hex	n/a				
3.97	OH-oxIAA hex-pent	C21H28NO13	502.1562	502.1561	0.2	424, 370, 208
4.09	OH-oxIAA hex-pent	C21H28NO13	502.1563	502.1561	0.4	424, 370, 208
4.32	MeO-oxIAA hex	C17H22NO9	384.1285	384.1295	-2.6	222, 190, 162, 144, 116
4.81	MeO-oxIAA hex-pent	C22H30NO13	516.1711	516.1717	-1.2	222, 190, 162, 144, 116
4.89	MeO-oxIAA hex-pent	C22H30NO13	516.1705	516.1717	-2.3	222, 190, 162, 144, 116
6.24	MeO-oxIAAe VA hex-hex-pent	C36H46NO21	828.2567	828.2562	0.6	666, 384, 283, 222, 190, 162, 144, 116
6.32	MeO-oxIAAe VA hex-hex-pent	C36H46NO21	828.2571	828.2562	1.1	666, 384, 283, 222, 190, 162, 144, 116
6.41	OH-oxIAA HBA hex-pent	C28H32NO15	622.1951	622.1925	4.2	n/a
6.50	OH-oxIAA HBA hex-pent	n/a				
6.59	OH-oxIAA VA hex-pent	n/a				
6.70	OH-oxIAA VA hex-pent	n/a				
6.96	MeO-oxIAAe FA hex-hex-pent	C38H48NO21	854.2765	854.2719	5.4	692, 365, 222, 177, 162, 144
7.06	MeO-oxIAAe FA hex-hex-pent	C38H48NO21	854.2702	854.2719	-2.0	692, 365, 222, 177, 162, 144
7.21	MeO-oxIAA HBA hex-pent	C29H34NO15	636.1937	636.1928	1.4	222, 190, 162, 144
7.34	MeO-oxIAA HBA hex-pent	C29H34NO15	636.1959	636.1928	4.9	222, 190, 162, 144
7.36	MeO-oxIAA VA hex-pent	C30H36NO16	666.2029	666.2034	-0.8	666, 384, 283, 222, 190, 162, 144, 116
7.48	MeO-oxIAA VA hex-pent	C30H36NO16	666.2041	666.2034	1.1	666, 384, 283, 222, 190, 162, 144, 116
7.65 (tr)	MeO-oxIAA VA hex-pent	n/a				
7.71 (tr)	MeO-oxIAA VA hex-pent	n/a				
7.83	OH-oxIAA FA hex-pent	n/a				

8.56	MeO-oxIAA FA hex-pent	C32H38NO16	692.2198	692.2191	1.0	617, 291, 222, 177, 144
8.63	MeO-oxIAA FA hex-pent	C32H38NO16	692.2193	692.2191	0.3	617, 291, 222, 177, 144
8.8 (tr)	MeO-oxIAA FA hex-pent	C32H38NO16	692.215	692.2191	-5.9	222, 177, 144
8.87 (tr)	MeO-oxIAA FA hex-pent	C32H38NO16	692.2161	692.2191	-4.3	222, 177, 144
9.31	MeO-oxIAA FA hex-pent-mal	C35H40NO19	778.2217	778.2195	2.8	748, 667, 222, 177, 162,144
9.36	MeO-oxIAA FA hex-pent-mal	C35H40NO19	778.2227	778.2195	4.1	222, 177, 162,144
9.74	unknown MeO-oxIAA conjugate	C32H42NO16	696.2488	696.2504	-2.3	222
9.82	unknown MeO-oxIAA conjugate	C32H42NO16	696.2505	696.2504	0.1	222
10.32	unknown MeO-oxIAA conjugate	C37H48NO16	762.3007	762.2973	4.5	222
10.39	unknown MeO-oxIAA conjugate	C37H48NO16	762.3044	762.2973	9.3	222
